# Human papillomavirus and *Chlamydia trachomatis* in oral and genital mucosa of women with normal and abnormal cervical cytology

**DOI:** 10.1186/s12879-021-06118-3

**Published:** 2021-05-05

**Authors:** J. P. Mosmann, S. Zayas, A. X. Kiguen, R. F. Venezuela, O. Rosato, C. G. Cuffini

**Affiliations:** 1 Facultad de Ciencias Médicas, Instituto de Virología “Dr. J. M. Vanella”, Universidad Nacional de Córdoba, Enfermera Gordillo Gómez s/n- Ciudad Universitaria, ZP: 5016 Córdoba, Argentina; 2 Consejo Nacional de Investigaciones Científicas y Técnicas (CONICET), Buenos Aires, Argentina; 3 Facultad de Ciencias Médicas, Hospital Universitario de Maternidad y Neonatología, Universidad Nacional de Córdoba, Córdoba, Argentina

**Keywords:** HPV, *Chlamydia trachomatis*, Cervical cytology, Infectious diseases, Cervical cancer

## Abstract

**Background:**

HPV and *C.trachomatis* are the most prevalent, viral and bacterial STI worldwide. Both commonly have an asymptomatic development and can evolve into a persistent infection which, added to coinfections, may be important cofactors for the oncogenic transformation.

**Objective:**

Evaluate the prevalence of oral and genital HPV and *C.trachomatis* infection in women with normal and abnormal cervical cytology.

**Study design:**

The cross-sectional study included 200 swabs, 100 oral and 100 cervical from 50 women with normal and 50 with abnormal cervical cytology. HPV and *C.trachomatis* infections were detected using PCR with specific primers.

**Results:**

HPV DNA was detected in 27% (*n* = 27/100) of women with normal and abnormal cytology. Out of 100 genital samples we detected HPV DNA in 18% (*n* = 18/100) and 14% (*n* = 14/100) out of 100 oral samples. HPV genotypes detected were genotype 6 of low-risk and 16, 31, 52, 58 and 16–31 coinfection of high-risk. *C.trachomatis* DNA was detected in 49% (*n* = 49/100) of patients. Out of 100 genital samples we detected *C.trachomatis* in 35% (*n* = 35/100) and 31% (*n* = 31) out of 100 oral samples. There is statistically significant (*p* < 0.05) between cytology and HPV and *C.trachomatis* infection but there is no statistically significant between cytology and the other characteristics.

**Conclusions:**

Since the histology of oral mucosa resembles that of the uterine cervix, we can anticipate the presence of HPV and other STI which are detected in different lesions of genital areas and the oral mucosa. Therefore, is important *C.trachomatis* detection and specific treatment in asymptomatic women because this infection may increase the risk of HPV persistence and coinfection induces a pro-inflammatory environment that may promote the carcinogenesis.

## Introduction

Human Papillomavirus (HPV) and *Chlamydia trachomatis* (*C.trachomatis*) are the most frequent, viral and bacterial respectively, sexually transmitted infections (STIs) worldwide and HPV is the necessary but no sufficient cause for cervical cancer [1]. Coinfections in cervical epithelium may be an important cofactor for the oncogenic transformation. There is evidence that *C.trachomatis* could act as a cofactor that may lead to epithelial disruption and facilitate HPV entry which facilitates HPV infection and contributes to the viral persistence, increasing the risk of developing cervical neoplasia [2, 3]. While screening strategies exist for cervical cancer prevention and the vaccination programs started, there is a lack of policies for the control of *C.trachomatis* infection [4]. *C.trachomatis* can cause different diseases such as cervicitis, endometritis, pelvic inflammatory disease and ectopic pregnancy [5].

Despite the well-established role of HPV in cervical cancer, evidence suggest that HPV may also be an independent risk factor for oral cancer [6] and it is proposed that *C.trachomatis* may be a cofactor for HPV associated oropharyngeal cancer [3, 7]. A potential correlation between genital and oral STIs suggests that oral sex may be the link to transmission from cervical to oral site [8]. The frequency of *C.trachomatis* in the oral cavity varies widely among published studies. This variability can be explained by the varied biological samples, the lack of global standardization techniques and the diversity of population study groups [9, 10].

Many risk factors such as age, tobacco, alcohol, hormonal contraceptive use and parity are associated with development cervical precancer and cancer, while cervical microenvironment, may also influence the natural history of HPV infection [3, 11, 12].

The present cross-sectional study aimed to evaluate the prevalence of oral and genital HPV and *C.trachomatis* infection in women with normal and abnormal cervical cytology.

## Materials and methods

### Study population

The cross-sectional study included oral and cervical swabs from 50 women with normal cervical cytology and 50 women with abnormal cervical cytology (Bethesda criteria), who had been referred to Maternity and Neonatology University Hospital- Argentina. Immunocompromised women were excluded. Patients included in the study, signed an informed consent form.

### Data and sample collection

A short questionnaire was used to collect data about age, number of sexual partners, oral and anal sex, number of pregnancies, history of STIs, oral lesion, contraceptive method, tobacco use and HPV vaccine.

The total number of cigarettes smoked throughout the patient’s life was calculated considering smoker to the person who smoked more than 100,000 units [13].

Cervical swabs were collected by a gynecologist using a brush and oral swabs were collected by a dentist, who did a bilateral scraping of oral cavity. Specimens were put in a sterile tube containing 1-ml phosphate buffer solution with antibiotic and antifungal agents to be processed. Both samples were collected by professional doctors on medical visit to Maternity and Neonatology University Hospital.

### Ethical approval

This study was approved by the Committee of Ethics, National Hospital of Clinics- Argentina (RePIS2548) according to the ethical principles stated in the declaration of Helsinki.

### HPV and *C.trachomatis* detection

DNA was extracted using the commercial AccuPrep Genomic DNA Extraction Kit-Bioneer, following the manufacturer’s instructions. HPV L1 genomic region (450 bp) was amplified with degenerate primers MY09 and MY11 following Manos’s protocol [14]. HPV-DNA positive samples were typed by restriction fragment length polymorphism method, using 7 restriction enzymes (BamHI, DdeI, HaeIII, HinfI, PstI, RsaI and Sau3AIII). This method allows us detect about 40 different HPV mucous genotypes, high risk genotypes: 16, 18, 31, 33, 35, 39, 45, 51, 52, 56, 58, 59; low risk: 6, 11, 13, 32, 40, 44, 55, 57, 61, 62, 64, 72, 81, 83, 84, 89 and intermediate risk: 26, 34, 42, 53, 54, 66, 67, 68, 70, 71, 73, 82 [15, 16]. *C.trachomatis* was detected using CTP1 and CTP2 primers to cryptic plasmid (201 bp) [17]. The PCR products were visualized by electrophoresis in 1.5% agarose. The β-globin gene was used as DNA preservation marker.

### Statistical analysis

Results were analyzed using Chi-square (X^2^) and Fisher Exact with a significance level 5% (95% CI), Epi info 3.5.4, CDC software (2012).

## Results

We studied 100 women who used the public health service, the women age range 18–67 years (mean 39). The women studied were divided according to normal and abnormal cytopathological findings. Normal papapanicolaou test include infections and inflammation and abnormal results includes atypical squamous cells of undetermined significance (ASCUS), low-grade squamous intraepithelial lesion (LSIL) and high-grade squamous intraepithelial lesion (HSIL) according to Bethesda system [18]. The normal group (NG) comprised 50 women who presented inflammatory cytology, whereas the abnormal group (AG) comprised 50 women, 9 with HSIL and 41 with LSIL. Table 1 shows the demographic, clinical, and molecular findings, and the relationship between the normal and abnormal status of the women in this study.

**Table 1:** Characteristics of patients studied according normal and abnormal cytology status

		Normal cytology		Abnormal cytology		
	N	N	%	N	%	***p***-value
**Total**	100	50	100	50	100	
**Age (years)**						0.516
**18–27**	2	1	2.0	1	2.0	
**28–37**	42	25	50.0	17	34.0	
**38–47**	40	15	30.0	25	50.0	
**48–57**	12	7	14.0	5	10.0	
**58–67**	4	2	4.0	2	4.0	
**Number of sexual partners**						0.450
**1 to 5**	84	44	88.0	40	80.0	
**6 to 10**	11	5	10.0	6	12.0	
**> 10**	4	1	2.0	3	6.0	
**Without data**	1	0	0.0	1	2.0	
**Oral sex**						0.094
**Yes**	66	29	58.0	37	74.0	
**No**	34	21	42.0	13	26.0	
**Anal sex**						0.629
**Yes**	33	16	32.0	17	34.0	
**No**	67	34	68.0	33	66.0	
**Pregnancies**						0.597
**0**	19	12	24.0	7	14.0	
**1 to 5**	78	36	72.0	42	84.0	
**6 to 10**	3	2	4.0	1	2.0	
**History of STIs**						0.387
**Yes**	6	2	4.0	4	8.0	
**No**	94	48	96.0	46	92.0	
**Oral lesion**						0.753
**Yes**	2	1	2.0	1	2.0	
**No**	98	49	98.0	49	98.0	
**Use of contraceptive method**						0.234
**Yes**	69	31	62.0	38	76.0	
**No**	31	19	38.0	12	24.0	
**Tobacco**						0.283
**Yes**	20	7	14.0	13	26.0	
**No**	80	43	86.0	37	74.0	
***C. trachomatis***						**0.013**
**Yes**	49	19	38.0	30	60.0	
**No**	51	31	42.0	20	40.0	
**HPV**						**0.041**
**Yes**	27	18	36.0	9	18.0	
**No**	73	32	64.0	41	82.0	
**Co-infection HPV-** ***C. trachomatis***						
**Yes**	14	7	14.0	7	14.0	1.000
**No**	86	43	86.0	43	86.0	

There is no statistically significance between normal and abnormal groups and risk factors such as age, number of sexual partners, oral sex, anal sex, pregnancies, history of STIs, oral lesion, contraceptive method and tobacco use (*p* > 0.05), Table 1. Reference to HPV vaccine, all women were asked about this, but only one of them was vaccinated (AG- LSIL, negative to HPV and *C.trachomatis*). Concerning history of STI, 2 patients with normal cytology had a history of *Trichomonas vaginalis* and Herpes Simplex Virus (HSV) infection. While in patients with abnormal cytology, 2 of them had a history of *C.trachomatis*, 1 of them HSV and 1 had history of *Treponema pallidum*.

**Fig. 1:**
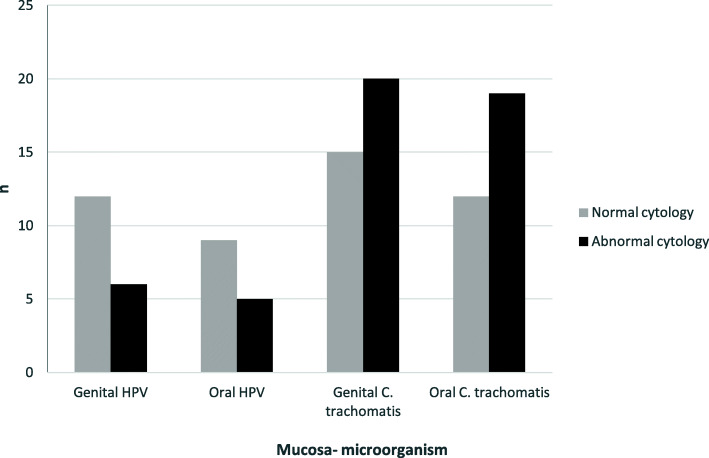
HPV and *C. trachomatis* distribution according cytology status in the both mucosa

**Fig. 2:**
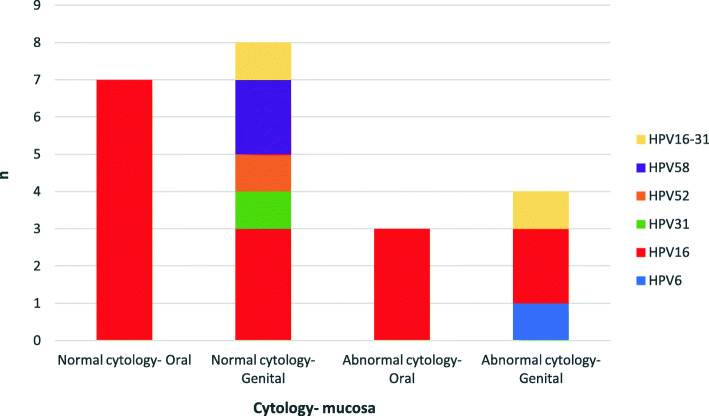
HPV genotypes detected according mucosa and cytology status

A total of 200 samples were studied, oral and cervical swabs were collected from each woman. HPV DNA was detected in 27% of women (27/100) with normal and abnormal cytology (Table 1). Out of 100 genital samples we detected HPV DNA in 18% (18/100) of these and out of 100 oral samples we detected HPV DNA in 14% (14/100); 5 patients with both mucosa infected- 3 NG and 2 AG (Fig. 1). There is no statistically significance between genital and oral HPV detection (*p* = 0,888). HPV genotypes detected were genotype 6 of low-risk and genotypes 16, 31, 52, 58 and 16–31 coinfection of high-risk. A total of 10 samples HPV DNA positive were genotype not identified. The more frequent genotype was the type 16 in normal and abnormal cytology as well as oral and genital mucosa. The only HPV type detected in oral mucosa was 16 (Fig. 2). Out of 5 women with oral and genital HPV, the same genotype was detected in 3 of them, all HPV 16. Two of them were not possible to obtain the genotype in one mucosa.

*C.trachomatis* DNA was detected in 49% of patients (49/100) (Table 1). Out of 100 genital samples we detected *C.trachomatis* in 35% (35/100) of these and out 100 oral samples we detect C.trachomatis DNA in 31% (31/100); 17 patients with both mucosa infected- 8 NG and 9 AG (Fig. 1). There is no statistically significance between genital and oral *C.trachomatis* detection (*p* = 0,732).

HPV was detected more frequent in NG (36%, 18/50) in comparison with AG (18%, 9/50), and in the different way *C.trachomatis* was detected more frequent in AG (60%, 30/50) in comparison with NG (38%, 19/50). There is statistically significance (*p* < 0.05) between cytology and HPV and *C.trachomatis* infection (Table 1), but there is no significance between HPV and *C.trachomatis* prevalence (*p* = 0.728).

We detected 14 (14%, 14/100) patients with the two infections, HPV and *C.trachomatis* (Table 2). Out of 14 patients, 7 had normal cytology and 7 had abnormal cytology.

**Table 2:** Characteristics of 14 patients coinfected with HPV and *C. trachomatis*

Cytology	Genital HPV	Oral HPV	HPV type	Genital ***C. trachomatis***	Oral ***C. trachomatis***
Abnormal- LSIL		✓	16	✓	✓
Abnormal- HSIL	✓		16–31	✓	✓
Abnormal- LSIL		✓	W/G		✓
Abnormal- LSIL	✓		W/G	✓	✓
Abnormal- LSIL		✓	W/G		✓
Abnormal- LSIL	✓		W/G	✓	
Abnormal- HSIL	✓	✓	16		✓
Normal	✓		W/G	✓	✓
Normal	✓		16–31	✓	
Normal	✓		W/G	✓	
Normal	✓		58	✓	
Normal		✓	16	✓	✓
Normal	✓	✓	16	✓	
Normal	✓		W/G		✓

*HSIL* High-grade squamous intraepithelial lesion, *LSIL* Low-grade squamous intraepithelial lesion, *W/G* Without genotype

## Discussion

When cervical HPV infection is investigated, different sites such as the oral cavity is not routinely investigated, except in the presence of visible lesions, as well neither *C.trachomatis* infection [19]. Although knowledge of HPV related tumor is clear, the prevalence of oropharyngeal HPV and *C.trachomatis* infection in unclear [8, 19]. Both microorganisms are important to public health, because existing chlamydial genital infection could increase the risk of acquiring HPV as well as favour viral persistent, leading to complications like cervical cancer [20].

The present results showed a total prevalence of 27% for HPV in the 100 patients studied. In reference to oral mucosa, we detect 14% of prevalence. Data about oral HPV, in asymptomatic patient is still on debate. Kreimer reported that asymptomatic oral HPV16 infection was found in 1.3% of a systematically reviewed and abstracted data from published studies (*n* = 18) that detected oral HPV DNA in 4581 cancer-free subjects [21] and Ciccarese detected 37% oral HPV DNA in women without signs of HPV infection who took part in the screening program on STI in Italy [22], both studies show a very different prevalence. In a previous local study in Córdoba-Argentina, HPV was not detected in oral mucosa without lesion or injury [11]. Likewise, in a study conducted in the same region among randomly selected healthy subjects, HPV was detected in 3% (13/401) and all the identified genotypes were low risk [23]. So, our prevalence detected is higher than these previous studies. Other study conducted by our group, detected 34% of oral HPV in patients with oral lesions (benign oral lesions, potentially malignant oral disorders and oral squamous cell carcinoma) [3], while in this present study only one patient had visible oral lesion. However, the absence of clinical signs of lesions in the oral cavity could show a subclinical infection which can be transmitted [19].

Concerning *C.trachomatis*, our results showed a 49% of prevalence. About oral *C.trachomatis*, we detect 31%. These results are higher in comparison with other study conducted by our group, in which we detected 17% *C.trachomatis* DNA positive in different oral lesions [3] but are similar to a study from Japanese population, in which 44% of *C.trachomatis* was detected in pharyngeal smears and 61% in oral fluid in sex workers [9]. This frequency was higher too than a study conducted in the Netherlands, which pharyngeal *C. trachomatis* was detected in 2.3% of women in free-of-charge and anonymous STI consultations on The STI outpatient clinic at the Public Health Service of Amsterdam [10] and higher than a review that described extra genital *C.trachomatis* infections, showed a prevalence of 0.2 to 3.2% in pharyngeal swabs from asymptomatic women [24]. It is important to highlight that the studies described above, had used different samples such as pharyngeal swab and oral fluid.

In reference to cytology status, we found a HPV detection rate of 36% in women with normal cytology and more prevalent in genital area. These results are in concordance with a study published from Brazil (Amazonian) in which a prevalence of 36.09% was detected in women with normal cytology who used public health services [25] and lower a retrospective study from Turkey, in which the prevalence of genital HPV in normal cytology was 49% in women with intact uterus with distinct gynecologic complaints [18]. Other study conducted in Brazil, showed a singular prevalence of HPV in women with normal cytology (27.2%) [26]. Patients with normal cytology tests and HPV HR types must be followed up because are at a high risk of having HPV induced lesions in the future [18].

In women with abnormal cytology we detected 18% HPV DNA positive, surprisingly prevalence lower than women with normal cytology but in agreement with Ji, who detected 18.4% HPV in women with abnormal cytology, who visited the department of gynecology of the hospital from China [5]. To difference with our results, Beyazit detected 51% of genital HPV in women with intact uterus with distinct gynecologic complaints [18], Ssedyabane 63.4% in patients that presented at the cervical cancer clinic [27] and a local study conducted by Venezuela detected 51.6% in women with abnormal cytology with antecedents of squamous intraepithelial lesions who attended private clinics and public health centers in Córdoba, Argentina [28]. When the patient had HPV positive test and normal cytology, a gynecological follow up is important to verify if HPV then is negative or if the cytology lesions has appeared [26].

Although HPV18 genotype is the second more frequent high risk type detected worldwide, in this study, we not detected HPV18 genotype in genital as well as oral mucosa. This is in agreeing with previous studies in oral mucosa in Argentina [3, 11, 23, 29]. Reference to genital mucosa, a Brazilian study not detected HPV18 genotype in normal mucosa [1, 25]. The more frequent HPV genotype detected was type 16; this result is in concordance with others studies in oral as well as genital mucosa [3, 5, 11, 18, 23, 25, 28, 29]. The coinfection detected in this work was high risk genotypes 16 and 31 in genital mucosa. However, previous reports suggest that HPV genotypes coinfection do not increase the risk of acquiring a new infection but may impair the immune response [5].

Concerning *C.trachomatis* and cytology status, we detect a prevalence of 38% in women with normal cytology and 60% in abnormal cytology, being more frequent in genital area. These results are in concordance with a study from India, which detected more prevalence of *C.trachomatis* in patients with abnormal cytology (31.5%) in comparison with normal cytology (5.8%) in women with complaints of vaginal discharge attending STI clinic in a tertiary care hospital in New Delhi [30]. On the other hand, other studies showed different results, such as local studies: Jordá, detected 8.5% *C.trachomatis* prevalence in symptomatic (vaginal discharge, pruritus, pelvis pain) and asymptomatic women with a request for a vaginal exudate study [31] and Kiguen, 6.9% in pregnant women, older than 14 years having 35 weeks of gestation [32], all results lower than our prevalence detected. Other studies, conducted by Ji, detected similar prevalence of *C.trachomatis* in women with normal and abnormal cytology, 7.1 and 7.2%, respectively, in China [5] and Costa Lira, detected to genital *C.trachomatis* in 9.02% in women who used public health services with abnormal cytology [25].

So, although some of our results, both HPV and *C.trachomatis*, are in concordance with previous result of our group and results of other authors, other prevalence are very different. The detection rate of HPV and *C. trachomatis* in the oral cavity specially, is known to vary widely in different places of the world. These differences could be associated with the different types of samples collected in each study, the type of detection technique used, the different habits of the populations studied, the oral pathologies present in the patients studied, and the prevalence and circulation microorganism of the area under study [33] As well as the prevalence of these agents in cervical lesions, it is influenced by the demographic and ethnic differences of the populations investigated as well as by the different diagnostic procedures [34].

The current study showed an association between cytology status and HPV and *C.trachomatis* infection, although HPV was more frequent in normal cytology. Therefore, *C.trachomatis* detection and specific treatment in asymptomatic women, is important to prevent future sequelae. However, some studies reported that *C.trachomatis* infection may increase the risk of HPV persistence and coinfection induces a pro-inflammatory environment that may promote the carcinogenesis [4]. In the present work, HPV/*C.trachomatis* coinfection was found in 14%, predominantly in genital mucosa. This result is in concordance to Costa Lira, who detected 12.5% of coinfection in cervical swabs of the women studied [25]. The presence of the pathogens in two anatomic sites could be for genetic predisposition or altered immune response. So, the viral concordance between the two anatomical sites appears not to be obligatory [19].

There are a lot of risk factors for STIs, specially for cervical lesions [6], however, in this study we not detected statistically significance between cytology status and the different risk factors studied.

## Conclusions

This study highlights the importance of HPV diagnosis and the sexually transmitted pathogens such as *C.trachomatis*, identified by many studies as possible cofactors for oncogenesis. There is evidence that suggest screening for *C.trachomatis* is cost effective when its prevalence is above 3% [35]. These results promote us to continue with investigation of important processes to patient health.

## Data Availability

The datasets used and/or analysed during the current study available from the corresponding author on reasonable request.

## Abbreviations

HPV: Human Papillomavirus
*C.trachomatis*: *Chlamydia trachomatis*
STI: Sexually Transmitted Infections
PCR: Polymerase Chain Reaction
DNA: Deoxyribonucleic Acid
Bp: Base pairs
ASCUS: Atypical squamous cells of undetermined significance
LSIL: Low-grade squamous intraepithelial lesion
HSIL: High-grade squamous intraepithelial lesion
NG: Normal group
AG: Abnormal group
